# Procalcitonin for the differential diagnosis of infectious and non-infectious systemic inflammatory response syndrome after cardiac surgery

**DOI:** 10.1186/2052-0492-2-35

**Published:** 2014-06-03

**Authors:** Dong Zhao, Jianxin Zhou, Go Haraguchi, Hirokuni Arai, Chieko Mitaka

**Affiliations:** Department of Critical Care Medicine, Beijing Tongren Hospital, Capital Medical University, Beijing, 100730 China; Department of Critical Care Medicine, Beijing Tiantan Hospital, Capital Medical University, Beijing, 100050 China; Department of Critical Care Medicine, Tokyo Medical and Dental University Graduate School, Tokyo, 113-8519 Japan; Department of Cardiovascular Surgery, Tokyo Medical and Dental University Graduate School, Tokyo, 113-8519 Japan

**Keywords:** Procalcitonin, Systemic inflammatory response syndrome, Cardiac surgery, Infection, C-reactive protein

## Abstract

**Background:**

This study was performed to assess the value of procalcitonin (PCT) for the differential diagnosis between infectious and non-infectious systemic inflammatory response syndrome (SIRS) after cardiac surgery.

**Methods:**

Patients diagnosed with SIRS after cardiac surgery between April 1, 2011 and March 31, 2013 were retrospectively studied. A total of 142 patients with SIRS, infectious (*n* = 47) or non-infectious (*n* = 95), were included. The patients with infectious SIRS included 11 with sepsis, 12 with severe sepsis without shock, and 24 with septic shock.

**Results:**

PCT, C-reactive protein (CRP), and the white blood cell (WBC) count were significantly higher in the infectious SIRS group than in the non-infectious SIRS group. PCT had the highest sensitivity and specificity for differential diagnosis, with a cut-off value for infectious SIRS of 0.47 ng/mL. PCT was more reliable than CRP in diagnosing severe sepsis without shock, but it was not useful for diagnosing septic shock. The PCT cut-off value for diagnosing severe sepsis without shock was 2.28 ng/mL.

**Conclusions:**

PCT was a useful marker for the diagnosis of infectious SIRS after cardiac surgery. The optimal PCT cut-off value for diagnosing infectious SIRS was 0.47 ng/mL.

## Background

According to an epidemiological survey, the incidence of sepsis in the USA rose at an average annual rate of 8.7% from 1979 to 2000 [1]. Sepsis and sepsis-related complications are the main causes of death in intensive care unit (ICUs). Of the approximately 750,000 patients with severe systemic infection reported every year in the USA, about 500 ultimately die of systemic infection and its complications [2]. Post-cardiac surgery patients make up a large proportion of patients treated in ICUs, and the incidence of systemic infections is high among them. According to one study, cardiac surgery has a mortality of 8.5% with infection and 2.2% without infection [3]. Early diagnosis and prompt treatment for infection will significantly improve the prognosis and avoid the unnecessary use of antibiotics for patients without infections [4]. It can still be very challenging, however, to reach an early differential diagnosis of infectious systemic inflammatory response syndrome (SIRS) based on clinical manifestations alone. Recent studies have shown a significant correlation of procalcitonin (PCT) with infection and suggest that PCT is useful for the early diagnosis of systemic infection [5, 6]. In the present study, we evaluated the differential diagnostic value of PCT for infectious SIRS after cardiac surgery and compared PCT with the two most traditional markers of infection, the C-reactive protein (CRP) and white blood cell (WBC).

## Methods

This study was a retrospective investigation of 142 SIRS patients who were admitted to the ICU after cardiac surgery at the Tokyo Medical and Dental University Hospital between April 1, 2011 and March 31, 2013 when a total of 376 patients after cardiac surgery were screened. The study was approved by the ethical review board of Tokyo Medical and Dental University Faculty of Medicine. The SIRS patients were divided into an infectious SIRS group and a non-infectious SIRS group according to the results of microbiological testing. The infectious SIRS group was further divided into three groups, namely, sepsis, severe sepsis without shock, and septic shock, according to the diagnostic criteria of the Surviving Sepsis Campaign Guidelines for Management of Severe Sepsis and Septic Shock: 2012 (SSCG2012) [7].

The pre-operation data, operation-related data, and post-operation data of both groups were analyzed and compared. The pre-operation data included the patient age, gender, body mass index (BMI), and concomitant diseases. The operation-related data included the cardiopulmonary bypass (CPB) duration, aortic cross clamping, operation time, and blood loss. The post-operation data were as follows: mechanical ventilation (MV) duration, disseminated intravascular coagulation (DIC) incidence, serum levels of PCT, CRP, and WBC, Acute Physiology and Chronic Health Evaluation (APACHE) II score, Sequential Organ Failure Assessment (SOFA) score, ICU stay, hospital stay, postoperative blood purification treatment ratio such as continuous renal replacement therapy (CRRT) and polymyxin B-immobilized fiber column-direct hemoperfusion (PMX-DHP), and postoperative extracorporeal membrane oxygenation (ECMO) therapy. DIC was identified using a score based on platelet count (>100 × 10^3^/μL = 0; <100 × 10^3^/μL = 1; <50 × 10^3^/μL = 2), elevated fibrin-related marker (e.g., soluble fibrin monomers or fibrin degradation products, no increase: 0; moderate increase: 2; strong increase: 3), prolonged prothrombin time (<3 s = 0; >3 but <6 s = 1; >6 s = 2), and fibrinogen level (>1.0 g/L = 0; <1.0 g/L = 1) derived from the International Society on Thrombosis and Haemostasis [8]. The total score of the four parameters ≥5 is compatible with overt DIC.

The APACHE II score and SOFA score were determined within the first 24 h from admission to the ICU. The PCT, CRP, and WBC measurements were conducted by a hospital test cabinet. The serum PCT level was determined with a ‘Cobas 6000’ analyzer (Roche; detection sensitivity of 0.05 ng/mL). The serum CRP level was determined by a latex coagulation detection method with a nephelometer. The PCT, CRP, and WBC levels measured on the first day after the diagnosis of SIRS in the ICU were used in this analysis.

### Statistical analysis

Statistical analyses were performed using a Statistical Package for Social Sciences (SPSS 17.0 Inc., Chicago, IL, USA) for Windows. For the numerical data, the homogeneity test of variance was done first. If the variance was homogeneous, the data were shown as mean ± SD. The numerical data comparisons between two groups were analyzed by the two-sided Student’s *t* test and comparisons of more than two groups were analyzed by the one-way analysis of variance test. If the variance was not homogeneous, the data were shown in median and interquartile ranges. Comparisons between two groups were done by the Mann-Whitney test and comparisons of more than two groups were done by the Kruskal-Wallis test. The categorical data comparisons between groups were analyzed by the Pearson Chi-square test or Fisher’s exact test. Statistical significance was assumed at *p* values of less than 0.05 on both sides. The abilities of PCT, WBC, and CRP to diagnose infection were evaluated by comparing the infectious and non-infectious groups by receiver operating characteristic (ROC) curve analyses. The abilities of PCT and CRP to diagnose severe sepsis without shock or septic shock were evaluated by ROC curve analyses comparing the sepsis group with the severe sepsis without shock/septic shock groups and comparing the severe sepsis without shock group with the septic shock group. The cut-off values for diagnosing infection, severe sepsis without shock, and septic shock were determined by the ROC curves. The diagnosis sensitivity, specificity, and positive and negative predictive values were calculated and compared.

## Results

### The characteristics of the infectious SIRS group and non-infectious SIRS group

In total, 142 patients were treated for SIRS after cardiac surgery. Among them, 47 were diagnosed with infectious SIRS and 95 were diagnosed with non-infectious SIRS. Table 1 shows the patients’ characteristics of the two groups. There were no significant differences between the two groups in age, gender, body mass index, CPB duration, aortic cross clamping, operative time, operative mortality, or blood loss. The hospital mortality, APACHE II score, SOFA score, MV duration, postoperative blood purification treatment ratio, postoperative extracorporeal membrane oxygenation (ECMO) therapy, DIC incidence, ICU stay, and hospital stay were significantly higher in the infectious SIRS group than in the non-infectious SIRS group. Three patients in the infectious SIRS group died of septic shock within 30 days after the operation, and another 19 patients died of septic shock 30 days after the operation during the hospital stay. The operative mortality and hospital mortality of the infectious SIRS group were 6.38% and 46.8%, respectively. Three patients in the infectious SIRS group were complicated with cerebral hemorrhage, and one was complicated with hepatic failure. No patient died during the hospital stay in the non-infectious SIRS group.

**Table 1:** **The comparison between the infectious and non-infectious groups**

	Infectious SIRS (***n*** = 47)	Non-infectious SIRS (***n*** = 95)	***p*** value
Age (year)	67.8 ± 15.7	67.2 ± 13.3	0.81
Gender, *n* (%)			0.77
Male	32 (68.1)	67 (70.5)	
Female	15 (31.9)	28 (29.5)	
Type of surgery, *n* (%)			
Valve surgery	16 (34.0)	36 (37.9)	0.65
CABG	9 (19.1)	11 (11.6)	0.22
Vessel replacement	13 (27.7)	21 (22.7)	0.47
VAD	3 (6.4)	1 (1.1)	0.11
Interventional therapy	1 (2.1)	14 (14.7)	0.02
Complex surgery	5 (10.6)	12 (12.6)	0.73
BMI, kg/m^2^	21.8 (20.0,26.7)	22.7 (20.4,24.7)	0.64
Concomitant disease, *n* (%)			
Diabetes mellitus	18 (38.3)	25 (26.3)	0.14
Hypertension	20 (42.6)	35 (36.8)	0.51
CKD	19 (40.4)	21 (22.1)	0.02
CPB duration, min	197.2 ± 142.5	173.6 ± 123.5	0.31
Aortic cross clamping, min	99.0 ± 100.1	98.3 ± 84.7	0.96
Operation time, min	508.9 ± 192.4	466.3 ± 174.9	0.19
Blood loss, mL	3,512 ± 3,150	3,189 ± 2,706	0.53
APACHE II	19.0 (15.0,25.0)	11.0 (9.0,14.0)	0.001
SOFA	7.0 ± 2.6	5.1 ± 2.1	0.001
PCT, ng/mL	2.80 (1.24,10.20)	0.10 (0.06,0.21)	0.001
WBC, ×10^3^/μL	15.5 (11.0,22.6)	9.3 (7.2,12.6)	0.001
CRP, mg/dL	15.8 (12.0,19.9)	5.8 (2.5,8.9)	0.001
ICU stay, day	23.0 (12.0,52.0)	5.0 (3.0,6.0)	0.001
Hospital stay, day	82.0 (37.0,149.0)	31.0 (23.0,45.0)	0.001
MV duration, h	264.0 (40.0,999.0)	17.0 (10.0,39.0)	0.001
CRRT, *n* (%)	26 (55.3)	5 (5.3)	0.001
PMX-DHP, *n* (%)	18 (38.3)	0	0.001
ECMO, *n* (%)	13 (27.7)	0	0.001
DIC, *n* (%)	23 (48.9)	0	0.001
30-Day mortality, *n* (%)	3 (6.38)	0	0.035
Hospital mortality, *n* (%)	22 (46.8)	0	0.001

*CABG* coronary artery bypass graft, *VAD* ventricular assist device, *BMI* body mass index, *CKD* chronic kidney disease, *CPB* cardiopulmonary bypass, *APACHE II* acute physiology and chronic health evaluation II, *SOFA* sequential organ failure assessment, *MV* mechanical ventilation, *CRRT* continuous renal replacement therapy, *PMX-DHP* polymyxin B-immobilized fiber column direct hemoperfusion, *ECMO* extracorporeal membrane oxygenation, *DIC* disseminated intravascular coagulation.

The infectious pathogens were Gram-positive bacteria in 19 patients, Gram-negative bacteria in 14 patients, and fungus in 16 patients. The positive culture specimens were sputum in 14, urine in 15, blood in 24, and drainage in 1.

### The differential diagnostic values of PCT for infectious and non-infectious SIRS

**Figure 1:**
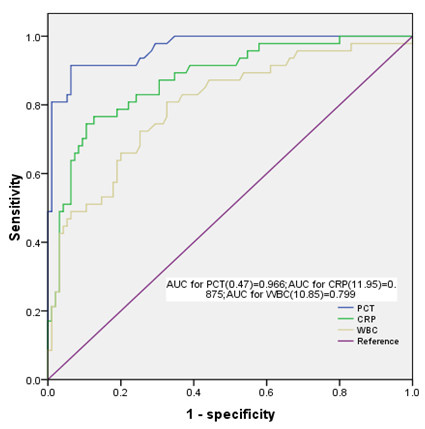
**The ROC curves of PCT, CRP, and WBC for predicting infection.**

PCT, CRP, and WBC levels were significantly higher in the infectious SIRS group than in the non-infectious SIRS group (Table 1). Preoperative chronic kidney disease (CKD), APACHE II and SOFA scores, ICU and hospital stay, MV duration, CRRT, PMX-DHP, ECMO therapy, DIC incidence, 30-day mortality, and hospital mortality were significantly higher in the infectious SIRS group than in the non-infectious SIRS group (Table 1). The ROC curves of PCT, CRP, and WBC for infectious SIRS are shown in Figure 1. The areas under the curve (AUC_ROC_) were 0.966, 0.875, and 0.799 for PCT, CRP, and WBC, respectively. According to the ROC curves, the cut-off values of PCT, CRP, and WBC were 0.47 ng/mL, 11.95 mg/dL, and 10.85 × 10^3^/μL, respectively. The sensitivity and specificity of PCT for predicting infection were 91.5% and 93.7%, respectively.

### Infection subgroup analysis

According to the diagnostic criteria, 47 patients from the infectious group were further divided into sepsis (*n* = 11), severe sepsis without shock (*n* = 12), and septic shock (*n* = 24). The patient characteristics of the three subgroups are shown in Table 2. No significant difference in WBC counts was found among the three subgroups, but the levels of PCT and CRP were significantly different. Preoperative CKD, CPB duration, APACHE II and SOFA scores, ICU and hospital stay, MV duration, CRRT, PMX-DHP, ECMO therapy, DIC incidence, and positive rate of MRSA culture were significantly higher in the septic shock subgroup than in the other two groups.

**Table 2:** **The comparison of infectious subgroups**

	Sepsis (***n*** = 11)	Severe sepsis without shock (***n*** = 12)	Septic shock (***n*** = 24)	***p*** value
Age (year)	70.6 ± 13.7	65.2 ± 19.0	67.8 ± 15.3	0.716
Gender, *n* (%)				0.058
Male	5 (45.5)	11 (91.7)	16 (66.7)	
Female	6 (54.5)	1 (8.3)	8 (33.3)	
BMI, kg/m^2^	21.6 (18.4,24.1)	20.6 (20.1,21.7)	23.9 (20.5,28.8)	0.090
Concomitant disease, *n* (%)				
Diabetes mellitus	3 (27.3)	6 (50.0)	9 (37.5)	0.531
Hypertension	5 (45.5)	3 (25.0)	12 (50.0)	0.351
CKD	4 (36.4)	2 (16.7)	13 (54.2)	0.001
CPB duration, min	102.0 ± 129.4	187.5 ± 134.8	245.6 ± 133.4	0.017
Aortic cross clamping, min	69.4 ± 92.4	108.8 ± 84.2	107.8 ± 111.4	0.542
Operation time, min	418.5 ± 136.1	540.8 ± 121.7	534.5 ± 231.9	0.206
Blood loss, mL	802 (398,2489)	6790 (1364,8324)	2530 (1227,4961)	0.019
APACHE II	15.5 ± 6.1	16.8 ± 7.6	23.5 ± 6.2	0.002
SOFA	5.5 ± 1.4	5.9 ± 2.9	8.2 ± 2.5	0.004
ICU stay, day	12.0 (4.0,23.0)	13.0 (7.3,24.5)	49.5 (17.8,81)	0.001
Hospital stay, day	60.9 ± 59.6	114.2 ± 85.5	124.5 ± 101.0	0.152
30-Day mortality, *n* (%)	0	0	3 (12.5)	0.215
Hospital mortality, *n* (%)	0	0	22 (91.7)	0.001
MV duration, h	40.0 (16.0,183.0)	58.5 (13.5,404.3)	992.5 (276.3,1559.3)	0.001
CRRT, *n* (%)	3 (27.3)	2 (16.7)	21 (87.5)	0.001
PMX-DHP, *n* (%)	0	0	17 (70.8)	0.001
ECMO, *n* (%)	0	1 (8.3)	12 (50.0)	0.001
DIC, *n* (%)	0	1 (8.3)	22 (91.7)	0.024
PCT, ng/mL	1.37 (0.72,1.85)	3.16 (0.48,13.24)	3.68 (1.67,20.96)	0.024
WBC, ×10^3^/μL	12.40 (9.10,24.20)	13.30 (9.93,16.93)	20.40 (13.45,28.6)	0.060
CRP, mg/dL	12.00 (7.40,18.00)	13.57 (8.17,18.13)	17.11 (15.15,30.6)	0.009
Gram positive, *n* (%)				
MRSA	0	0	6 (25.0)	0.037
*S. epidermidis*	2 (18.7)	4 (33.3)	1 (4.2)	0.064
*E. faecalia*	0	0	3 (12.5)	0.215
*C. striatum*	0	0	3 (12.5)	0.215
Gram negative, *n* (%)				
*S. marcescens*	0	2 (16.7)	2 (8.3)	0.359
*P. aeruginosa*	1 (9.1)	1 (8.3)	3 (12.5)	0.913
*B. cepacia*	1 (9.1)	1 (8.3)	1 (4.2)	0.815
*K. pneumoniae*	1 (9.1)	1 (8.3)	0	0.335
*Candida*, *n* (%)				
*C. albicans*	4 (36.4)	2 (16.7)	3 (12.5)	0.242
*C. glabrata*	0	0	2 (8.3)	0.368
*C. parapsilosis*	0	1 (8.3)	1 (4.2)	0.613
*C. tropicalis*	1 (9.1)	1 (8.3)	1 (4.2)	0.815

*MRSA* methicillin-resistant *Staphylococcus aureus*; for other abbreviations, see Table 1.

### Differential diagnostic values of PCT for the severity of sepsis

**Figure 2:**
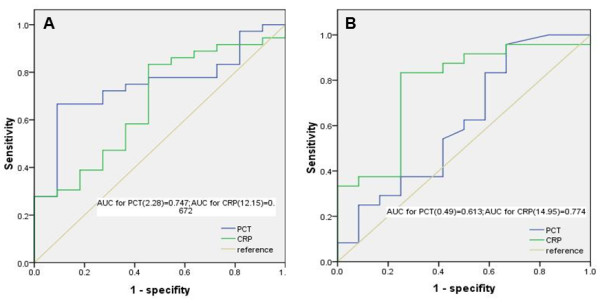
**ROC curves of PCT and CRP for predicting (A) severe sepsis without shock and (B) septic shock.**

The ROC curves of PCT and CRP for diagnosing severe sepsis without shock and septic shock are shown in Figure 2. PCT could be used for diagnosing severe sepsis without shock with a larger AUC than CRP. PCT was not useful, however, for diagnosing septic shock. The PCT cut-off value for diagnosing severe sepsis without shock was 2.28 ng/mL, with a sensitivity and specificity of 66.7% and 90.9%, respectively. The positive predictive value and negative predictive value of PCT for severe sepsis without shock were 96.0% and 45.5%, respectively. The cut-off value of CRP for diagnosing septic shock was 14.95 mg/dL, with a sensitivity and specificity of 83.3% and 66.7%, respectively. The positive predictive value and negative predictive value of CRP for septic shock were 87.0% and 69.2%, respectively.

## Discussion

PCT is a calcitonin precursor with 116 amino acids, no hormonal activity, and a relative molecular weight of 13,000. PCT is mainly secreted by the thyroid C-cells and peripheral mononuclear cells and has a half-life of about 24 h. PCT levels are extremely low and often difficult to detect in healthy people. At the time of infection, a bacterial component such as lipopolysaccharide or a cytokine such as tumor necrosis factor-α (TNF-α) or interleukin-6 (IL-6) induces high expression of the calcitonin-I gene, which activates the continuous release of PCT [9].

The surgical injury to the body and extracorporeal circulation during cardiac surgery can activate the complement system, which in turn induces the massive release of inflammatory cytokines and the subsequent onset of SIRS [10]. Once developed, SIRS goes on to cause multiple tissue and organ dysfunction, leading to a variety of postoperative complications [3]. Patients who elude SIRS from operative injuries still face the risk of SIRS induced by postoperative infection. Early diagnosis and prompt treatment of infection significantly improve the prognosis. Yet the differential diagnosis of infectious and non-infectious SIRS from different causes is still challenging. Some investigators have been convinced of the diagnostic value of PCT for differentiating infectious SIRS from non-infectious SIRS [11–14]. According to their reports, PCT indicates infection more reliably than traditional indicators such as body temperature, WBC, CRP, or cytokines (IL-6, IL-8). Yet another study has concluded that PCT was poor at distinguishing infectious SIRS from non-infectious SIRS [15]. In the present study, we found PCT to be useful in the diagnosis of infectious SIRS after cardiac surgery, and to have a higher sensitivity and specificity than any of the traditional markers. We suspect that the discrepancies in the results on PCT are linked to the many conditions that can affect PCT secretion. Infection, surgery, trauma, and other stress factors unrelated to infection can elevate PCT levels to varying degrees. The patients studied have also been quite heterogeneous; hence the PCT cut-off values for diagnosis have varied widely based on different diseases or different degrees of stress. As factors unrelated to infection, the preoperative CKD may affect PCT levels because of decreased renal elimination. Contou et al. have shown that using the threshold of 0.85 ng/mL PCT was a strong independent predictor of infection [16], suggesting that the PCT threshold was rising a little in CKD. On the other hand, Maisner et al. have reported that PCT levels were unchanged during CRRT in sepsis patients with acute kidney injury, although elimination of PCT depended on the duration of CRRT [17]. PMX-DHP may also affect the PCT levels, since it adsorbs endotoxin and decreases inflammation. However, PMX-DHP was not started at the time of PCT measurement. Stress factors such as ECMO therapy may affect PCT levels. Rungatscher et al. have shown that high levels of PCT were associated with multiple organ dysfunction syndrome in pediatric patients with ECMO [18]. This finding is consistent with our result that PCT levels increased in proportion to the severity of sepsis and multiple organ dysfunction syndrome. Consequently, PCT levels in the infectious SIRS group were significantly (*p* < 0.001) higher than those in the non-infectious SIRS group. Therefore, PCT becomes a tool to distinguish infectious and non-infectious SIRS, although some factors such as CKD and multiple organ dysfunction syndrome may affect the PCT level.

Clec’h et al. reported a wide variability of PCT cut-off values in an analysis of 143 patients with different diseases [19]. By their calculation, the optimal PCT cut-off value for diagnosing infection in surgical patients was 9.7 ng/mL (sensitivity 91.7%, specificity 74.2%), whereas the optimal PCT cut-off value for infection diagnosis in non-surgical patients was 1.00 ng/mL (sensitivity 80%, specificity of 94%). Meisner et al. investigated the correlations of operative techniques with the PCT cut-off values. By their estimate, the optimal PCT cut-off value in diagnosis of infection in patients after cardiac surgery was 2.0 ng/mL [20]. In describing PCT as a marker of infection, the aforesaid two studies recommended that the cut-off value be set according to the patient’s diagnosis and the type of surgery performed. PCT had a better diagnostic value than CRP and WBC in the present study. For the patients after cardiac surgery, the optimal PCT cut-off value for diagnosing infectious SIRS was 0.47 ng/mL. The sensitivity and specificity of PCT for the differential diagnosis of infectious SIRS and non-infectious SIRS at this cut-off (91.5% and 93.7%, respectively) were significantly better than those of CRP and WBC. When comparing the cut-off values, we should consider both the primary disease and type of surgery, as well as the time point of markers measurement.

The utility of PCT for evaluating infection severity has also been controversial. One study suggested that the PCT was a valuable parameter for determining infection, but was no better than CRP in evaluating the infection severity [21]. In contrast, Harbarth et al. found that serum PCT concentrations increased in step with the severity of infection [11]. Our results, like those of Harbarth’s group, demonstrated a rise in serum PCT concentrations in association with infection severity. The median PCT concentrations in sepsis, severe sepsis without shock, and septic shock group were 1.37, 3.16, and 3.68 ng/mL, respectively (Table 2). PCT was a good marker of severe sepsis without shock (cut-off value of 2.28 ng/mL), but it had no significant value for diagnosing septic shock. This was consistent with the study by Brunkhorst et al. [22].

The MV duration, postoperative blood purification therapy, postoperative ECMO therapy, DIC incidence, ICU stay, and hospital mortality were all significantly higher in the infectious SIRS group than in the non-infectious SIRS group. These findings indicate that infection will increase the disease severity and necessitate more invasive treatments such as MV, CRRT, and ECMO. This finding was consistent with the results of Rahmanian et al. [23]. The septic shock subgroup in the present study had more blood infections, especially MRSA infections. This finding was consistent with the study by Chen et al. [24].

There are three limitations to this study worthy of note: The study was retrospective; the samples were too small, especially for the infectious group; and no long-term follow-up was performed.

## Conclusions

Infection after cardiac surgery significantly increased the disease severity and necessitated more invasive treatments such as mechanical ventilation, CRRT, and ECMO. PCT was a useful marker for the diagnosis of infectious SIRS after cardiac surgery. PCT had a better diagnostic value than CRP or WBC. The optimal PCT cut-off value for detecting infection was 0.47 ng/mL. The serum level of PCT rose significantly according to the degree of infection. Prospective, large-scale, controlled, and randomized studies are awaited.

## Abbreviations

APACHE II: acute physiology and chronic health evaluation II
AUC: area under the curve
CRP: C-reactive protein
CRRT: continuous renal replacement therapy
CKD: chronic kidney disease
DIC: disseminated intravascular coagulation
ECMO: extracorporeal membrane oxygenation
ICU: intensive care unit
IL: interleukin
PCT: procalcitonin
PMX-DHP: polymyxin B-immobilized fiber column direct hemoperfusion
SIRS: systemic inflammatory response syndrome
SOFA: sequential organ failure assessment
TNF-α: tumor necrosis factor-α
ROC: receiver operating characteristic
WBC: white blood cell.
